# Supplementary material for: Post-Herpetic Anti-NMDAR Encephalitis in Denmark: Current Status and Future Challenges
Source: Biomedicines. 2024 Aug 27;12(9):1953. doi: 10.3390/biomedicines12091953 (PMC11429063; doi:10.3390/biomedicines12091953)
Supplement: Supplementary file 1 [file biomedicines-12-01953-s001.zip › biomedicines-3105203-supplementary.pdf]

Supplementary

Supplementary Table S1| Demographics and references for the 80 cases with post-HSE NMDARE in the literature.

Patient no	1	2	3	4	5	6	7	8	9	10	11	12	13	14	15	16
Sex	F	F	F	F	M	M	F	M	F	M	F	M	M	M	M	M
Age (years)	58	0,83	0,75	2	0,17	41	45	82	0,5	0,67	34	45	50	15	13	2,25
Reference	[1]	[1]	[1]	[2]	[3]	[16]	[16]	[16]	[16]	[16]	[4]	[4]	[4]	[4]	[4]	[5]
Patient no	17	18	19	20	21	22	23	24	25	26	27	28	29	30	31	32
Sex	F	M	M	M	F	M	F	F	F	F	M	F	M	M	M	M
Age (years)	58	60	0,25	0,42	0,5	0,58	0,58	0,67	0,67	0,75	0,92	0,92	0,92	1	1,25	1,25
Reference	[5]	[5]	[5]	[5]	[5]	[5]	[5]	[5]	[5]	[5]	[5]	[5]	[5]	[5]	[5]	[5]
Patient no	33	34	35	36	37	38	39	40	41	42	43	44	45	46	47	48
Sex	F	F	F	M	F	F	M	F	M	M	F	M	M	F	M	F
Age (years)	1,33	1,42	1,63	2,17	2,17	2,25	4	6	7	10	12	12	17	33	46	51
Reference	[5]	[5]	[5]	[5]	[5]	[5]	[5]	[5]	[5]	[5]	[5]	[5]	[5]	[5]	[5]	[5]
Patient no	49	50	51	52	53	54	55	56	57	58	59	60	61	62	63	64
Sex	M	F	F	M	F	M	M	F	M	M	F	F	F	F	M	F
Age (years)	58	1,33	1,58	35	33	-	71	57	3	0,46	1,25	1,33	3	15	2	0,67
Reference	[5]	[6]	[7]	[8]	[9]	[10]	[11]	[31]	[12]	[13]	[13]	[13]	[14]	[3]	[15]	[16]
Patient no	65	66	67	68	69	70	71	72	73	74	75	76	77	78	79	80
Sex	M	F	F	F	M	F	M	M	F	F	F	M	M	F	F	F
Age (years)	7	67	7	3	0,75	84	30	24	44	64	46	53	61	52	0,92	0,5
Reference	[32]	[17]	[18]	[19]	[20]	[21]	[22]	[23]	[23]	[23]	[23]	[23]	[24]	[25]	[26]	[31]

- = age unknown

Supplementary Table S2 | Detailed overview of HSE patients in the Region of Southern Denmark. Patients were divided into those with a relapsing/prolonged HSE disease course or without.

	All	Relapse/prolonged		P-value
	(*n=44*)	Yes (*n=8*)	No (*n=36*)	
Demographics				
Sex, male, % (n/N)	52.3 (23/44)	62.5 (5/8)	50.0 (18/36)	0.701
Age at diagnosis, years, median (range)	69.5 (25-88)	71.0 (35-87)	69.5 (25-88)	0.648
Symptoms				
Temperature, °C , median (range)	39.0 (36.2-40.6)	39.0 (36.2-39.6)	39.0 (36.4-40.6)	0.738
Fever (≥38.0 °C), % (n/N)	72.7 (32/44)	75.0 (6/8)	72.2 (26/36)	1.000
Decreased consciousness (GCS≤13), % (n/N)	36.4 (16/44)	75.0 (6/8)	27.8 (10/36)	0.019
GCS at admission, median (range)	14 (3-15)	13.5 (7-15)	14 (3-15)	0.256
Lowest GCS, median (range)	10 (3-15) (*n=43*)	7 (3-14)	10 (3-15) (*n=35)*	0.458
Cognitive impairment[a], % (n/N)	95.3 (41/43)	100 (8/8)	94.3 (33/35)	1.00
Speech abnormalities[b], % (n/N)	65.9 (27/41)	87.5 (7/8)	60.6 (20/33)	0.227
New focal symptoms, % (n/N)	37.2 (16/43)	37.5 (3/8)	37.1 (13/35)	1.00
Personality changes[c], % (n/N)	53.5 (23/43)	50 (4/8)	54.3 (19/35)	1.00
Seizures, % (n/N)	52.3 (23/44)	37.5 (3/8)	55.6 (20/36)	0.448
Abnormal movements[d], % (n/N)	13.6 (6/44)	12.5 (1/8)	13.9 (5/36)	1.00
Hallucinations, % (n/N)	30.0 (12/40)	37.5 (3/8)	28.1 (9/32)	0.677
Work diagnosis, % (n/N)				
Apoplexia + TIA	36.4 (16/44)	25 (2/8)	37.8 (14/36)	0.689
Meningitis	13.6 (6/44)	12.5 (1/8)	13.5 (5/36)	1.00
Encephalitis	4.5 (2/44)	12.5 (1/8)	2.7 (1/36)	0.334
Infection / sepsis	29.5 (13/44)	12.5 (1/8)	32.4 (12/36)	0.402
Seizure/epilepsy	4.5 (2/44)	0 (0/8)	5.4 (2/36)	1.00
Other[e]	11.4 (5/44)	37.5 (3/8)	5.4 (2/36)	0.035
CSF				
Leucocytes, median (range)	69 (2.5-790)	110 (5-260)	50.5 (2.5-790)	0.594
Protein, median (range)	0.55 (0.27-6.12) (*n=40)*	0.75 (0.27-2.45) (*n=6)*	0.55 (0.3-6.12) (*n=34)*	0.519
Abnormal IgG index or OCB, % (n/N)	40 (4/10)	33.3 (1/3)	42.9 (3/7)	1.00
MRI, % *(n/N)*				
Bilateral hyperintensity	30.2 (13/43)	25 (2/8)	31.4 (11/35)	1.00
Unilateral hyperintensity	51.2 (22/43)	50 (4/8)	51.4 (18/35)	1.00
Normal or unspecific	20.9 (9/43)	25 (2/8)	20.0 (7/35)	1.00
EEG, % *(n/N)*				
Paroxcystic	54.8 (23/42)	85.7 (6/7)	48.6 (17/35)	0.105
Focal slowing	92.9 (39/42)	100 (7/7)	91.4 (32/35)	1.00
Generalized slowing	4.8 (2/42)	0 (0/7)	5.7 (2/35)	1.00
Normal or unspecific	2.4 (1/42)	0 (0/7)	2.9 (1/35)	1.00
Timeline, median days (range)				

Symptoms to admission	2 (0-21) *(n=42)*	3.5 (0-14)	2 (0-21) *(n=34)*	0.746
Symptoms to diagnosis	5.5 (1-76) *(n=42)*	7 (2-76)	5 (1-47) *(n=34)*	0.215
Symptoms to treatment	4 (0-29) *(n=43)*	4 (0-29)	4 (0-22) *(n=35)*	0.582
Treatment duration	16 (4-30) *(n=43)*	20.5 (10-24)	15 (4-30) *(n=35)*	0.206
Admission length	21 (6-82)	23 (15-52)	21 (6-82)	0.420
Symptomatic Treatment, % *(n/N)*				
AED	60.5 (26/43)	75 (6/8)	57.1 (20/35)	0.446
Glucocorticoid treatment	20.5 (9/44)	0.0 (0/8)	25 (9/36)	0.175
ICU	31.8 (14/44)	25 (2/8)	33.3 (12/36)	1.00
Criteria, %*(n/N)*				
Probable NMDAR AE	34.1 (15/44)	50 (4/8)	30.6 (11/36)	0.414
Definite NMDAR AE	0 (0/44)	0 (0/8)	0 (36/36)	NA
Death before discharge, %	20.5 (9/44)	0 (0/8)	25 (9/36)	0.175

[a]Memory malfunction and confusion, [b]Aphasia, pressured speech, verbal reduction or mutism, [c]Personality changes or abnormal behavior, [d]Dyskinesia, [e]Intoxication *(n=2)*, arthritis temporalis *(n=1)*, depression *(n=1)*, dehydration (n=2). Abbreviations: *Ab*, Antibodies; *AE,* Autoimmune Encephalitis; *AED,* Antiepileptic Drugs; *EEG*, Electroencephalography; eGOS, estimated Glasgow outcome score; GCS, Glasgow coma scale; MRI = magnetic resonance imaging; NMDAR, *N*-Methyl-D-Aspartate receptor; *OCB*, Oligoclonal Bands; *ICU*, Intensive Care Unit; *TIA*, Transitory Ischemic Attack

Supplementary Table S3 | General clinical and paraclinical features at first admission vs. readmission in the eight adult HSE patients identified with relapse/prolonged course of disease, Region of Southern Denmark 2009-2021

	First admission	Relapse/prolonged admission
Symptoms in overall disease course		
Temperature, ◦C, median (range)	39.0 (36.2-39.6)	37.3 (36.4-39.2) *(n=7)*
Decreased consciousness (GCS≤13), % (n/N)	75 (6/8)	37.5 (3/8)
GCS at admission, median (range)	13.5 (7-15)	14.5 (6-15)
GCS lowest during admission, median (range)	7 (3-14)	13 (6-15) *(n=7)*
Cognitive impairment, % (n/N)	100 (8/8)	87.5 (7/8)
Speech abnormalities, % (n/N)	87.5 (7/8)	62.5 (5/8)
New focal symptoms, % (n/N)	37.5 (3/8)	37.5 (3/8)
Personality changes, % (n/N)	50 (4/8)	62.5 (5/8)
Seizures, % (n/N)	37.5 (3/8)	75 (6/8)
Abnormal movements, % (n/N)	12.5 (1/8)	25 (2/8)
Hallucinations, % (n/N)	37.5 (3/8)	25 (2/8)
Work diagnosis, % (n/N)		
Apoplexy + TIA	25 (2/8)	0 (0/7)
Meningitis	12.5 (1/8)	0 (0/7)
Encephalitis	12.5 (1/8)	0 (0/7)
Infection / sepsis	12.5 (1/8)	0 (0/7)
Seizure/epilepsy	0 (0/8)	12.5 (1/7)
Other	37.5 (3/8)	12.5 (1/7)
Relapse HSE	-	57.1 (4/7)
Sequelae after HSE	-	12.5 (1/7)
CSF		
Leucocytes, median (range)	110 (5-260)	20 (12-43) *(n=5)*
Protein, median (range)	0.75 (0.27-2.45) *(n=6)*	0.88 (0.27-1.19) *(n=6)*
OCB or IgG index, % (n/N)	33.3 (1/3)	75 (3/4)
Tested for NMDAR-ab, % (n/N)	25 (2/8)	50 (4/8)
Tested positive for NMDAR-ab, % (n/N)	0 (0/2)	75 (3/4)
MRI, % (n/N)		
Bilateral hyper intensity	25 (2/8)	60 (3/5)
Unilateral hyper intensity	50 (4/8)	20 (1/5)
Normal or unspecific	25 (2/8)	20 (1/5)
EEG, % (n/N)		
Paroxystic	85.7 (6/7)	20 (1/5)
Focal slowing	100 (7/7)	60 (3/5)
Generalized slowing	0 (0/7)	40 (2/5)
Normal or unspecific	0 (0/7)	0 (0/5)
